# Long-term continuous renal replacement therapy and anticoagulation with citrate in critically ill patients with severe liver dysfunction

**DOI:** 10.1186/s13054-017-1870-3

**Published:** 2017-11-29

**Authors:** Matthias Klingele, Theresa Stadler, Danilo Fliser, Timo Speer, Heinrich V. Groesdonk, Alexander Raddatz

**Affiliations:** 1 Department of Internal Medicine – Nephrology and Hypertension, Saarland University Medical Centre, Homburg/Saar, Germany; 2 Departments of Nephrology and Internal Medicine, Hochtaunus-Kliniken, Zeppelinstrasse 20, D-61352 Bad Homburg, Germany; 3 Departments of Nephrology and Internal Medicine, Hochtaunus-Kliniken, 61250 Usingen, Germany; 4 Department of Anaesthesiology, Intensive Care Medicine and Pain Medicine, Saarland University Medical Centre, Homburg/Saar, Germany

**Keywords:** CRRT, Anticoagulation, Citrate, Critically ill, Liver, Accumulation

## Abstract

**Background:**

As of 2009, anticoagulation with citrate was standard practice in continuous renal replacement therapy (CRRT) for critically ill patients at the University Medical Centre of Saarland, Germany. Partial hepatic metabolism of citrate means accumulation may occur during CRRT in critically ill patients with impaired liver function. The aim of this study was to evaluate the actual influence of hepatic function on citrate-associated complications during long-term CRRT.

**Methods:**

In a retrospective study conducted between January 2009 and November 2012, all cases of dialysis therapy performed in the interdisciplinary surgical intensive care unit were analysed. Inclusion criteria were CRRT and regional anticoagulation with citrate, pronounced liver dysfunction, and pathologically reduced indocyanine green plasma disappearance rate (ICG-PDR).

**Results:**

A total of 1339 CRRTs were performed in 69 critically ill patients with liver failure. At admission, the mean Model for End-stage Liver Disease score was 19.2, and the mean ICG-PDR was 9.8%. Eight patients were treated with liver replacement therapy, and 30 underwent transplants. The mortality rate was 40%. The mean duration of dialysis was 19.4 days, and the circuit patency was 62.2 h. Accumulation of citrate was detected indirectly by total serum calcium/ionised serum calcium (tCa/iCa) ratio > 2.4. This was noted in 16 patients (23.2%). Dialysis had not to be discontinued for metabolic disorder or accumulation of citrate in any case. In 26% of cases, metabolic alkalosis occurred with pH > 7.5. Interestingly, no correlation between citrate accumulation and liver function parameters was detected. Moreover, most standard laboratory liver function parameters showed poor predictive capabilities for accumulation of citrate.

**Conclusions:**

Our findings indicate that extra-hepatic metabolism of citrate seems to exist, avoiding in most cases citrate accumulation in critically ill patients despite impaired liver function. Because the citric acid cycle is oxygen-dependent, disturbed microcirculation would result in inadequate citrate metabolism. Raising the tCa/iCa ratio would therefore be an indicator of severity of illness and mortality rather than of liver failure. However, further studies are warranted for confirmation.

## Background

Acute renal failure requiring dialysis is a common complication in critically ill patients in intensive care units (ICUs) [1, 2]. In order to perform renal replacement therapy (RRT), adequate anticoagulation is necessary. Heparin is often used for this purpose [2]. However, the bleeding risk may be elevated in critically ill patients, especially in the context of surgery or patients with hepatic impairment. Regional anticoagulation with citrate lowers the risk of bleeding complications and therefore appears to be advantageous in these patients [3]. Admitted citrate is half-eliminated during dialysis. The residual half of citrate does return to the patient. Citrate metabolism is oxygen-dependent via the citric acid cycle and therefore mainly in organs with high amounts of mitochondria, such as the liver, kidney or muscle [4]. In the literature, citrate metabolism is described as being dependent mainly on liver function [5, 6]. Thus, regional anticoagulation with citrate is considered critical in cases of liver dysfunction with respect to the potential risk of citrate accumulation resulting in metabolic disorders, including shifts in calcium balance [6]. Because of these potential side effects, regional anticoagulation with citrate is often avoided in patients with hepatic insufficiency and mostly excluded in studies investigating regional anticoagulation with citrate [7]. To this day, there is still little knowledge about the actual incidence of citrate-associated complications in patients with impaired liver function. Moreover, the existing rare studies describe only short periods of dialysis [8, 9]. Information about continuous renal replacement therapy (CRRT) of longer duration with citrate anticoagulation in critically ill patients with hepatic insufficiency is not available.

This study had three aims: to evaluate the proportion of critically ill patients with severe hepatic insufficiency developing citrate-associated complications during CRRT, to investigate the actual influence of hepatic impairment and duration of CRRT on the development of these citrate-associated complications, and finally to identify predicting factors for citrate-associated complications in critically ill patients with impaired liver function.

## Methods

### Study cohort

Since January 2009, regional anticoagulation with citrate is routinely used for RRT in all ICUs at the Saarland University Medical Centre. From that time until November 2012, all dialysis cases in the interdisciplinary surgical ICU were analysed. The ethics committee of the medical association evaluated this retrospective study of anonymised data and voted that no ethical approval or consent to participate was needed (ethics vote of the Ethik-Kommission der Aerztekammer des Saarlandes [211/11]).

Inclusion criteria were CRRT and anticoagulation with citrate, pre-existing severe liver dysfunction, and/or developing acute liver failure during the clinical course in the ICU. Patients with known cirrhosis, elevated bilirubin or ammonia, and/or reduced synthesis parameters (e.g., prothrombin time [expressed as Quick value], cholinesterase [CHE], albumin) were considered as showing chronic and/or acute liver dysfunction. Because indocyanine green plasma disappearance rate (ICG-PDR) is a widely accepted parameter for clinical assessment of liver function and is normally > 18%/minute [10–12], only patients in whom ICG-PDR had been performed were included. Unfortunately, ICG-PDR was not determined systematically in all patients with signs of liver dysfunction. Primary endpoints were metabolic disorders due to citrate.

The aim of this study was to investigate the influence of liver function on the metabolism of citrate during long-term continuous venovenous haemodialysis (CVVHD) in critically ill patients. We therefore focus on data characterising our study population regarding pre-existing liver disease, severity of illness and outcome parameters.

All clinical data were obtained by assessing patient medical records. Laboratory parameters taken for clinical monitoring were processed within the central laboratory of the Saarland University Medical Centre. Owing to the retrospective study design, not all laboratory data were available at all time points desired for this evaluation.

During the study period, Simplified Acute Physiology Score II (SAPS II) and Therapeutic Intervention Scoring System score were automatically determined every day. At this time point, the more often used Sepsis-related Organ Failure Assessment score was not automatically or routinely determined.

### CRRT and anticoagulation with citrate

CRRT was performed with multifiltrate CiCa dialysate (Fresenius Medical Care, Bad Homburg, Germany) as part of the regional citrate anticoagulation (RCA). Post-filter-ionised calcium levels were used for anticoagulation monitoring. The concentration of post-filter-ionised calcium (iCa) was reduced to 0.25–0.35 mmol/L by chelation with citrate as described by Calatzis and colleagues [13]. Post-filter-ionised calcium levels were measured three times within the first 2 h after initiation of CRRT. After achieving stable iCa within the target range, post-filter levels of iCa were measured at least four times per day. Calcium was added before the blood was returned into the patient’s circulation to achieve a level within the normal range (2.2–2.6 mmol/L). Serum levels of albumin-corrected total serum calcium (tCa) were measured at least once per day.

CVVHD was the only renal replacement modality used. All CRRTs were started according to our standard protocol: Initial blood flow was 100 ml/minute, dialysate flow was 2000 ml/h, citrate solution was infused pre-filter at an initial rate of 4.0 mmol/L blood, and calcium was substituted with 1.7 mmol/L dialysate. In patients with a body weight > 80 kg, flow rates of blood and dialysate were likewise increased as target dialysis dose was > 25 ml/kg/h.

In case of accumulation of citrate, in a first step, dialysate flow was increased by 20–25%. In a second step, blood flow was reduced by 10–20%, if possible with respect to the body weight (minimal blood flow, 1 ml/minute/kg body weight) and target dialysis dose. The last step for correction was a further increase in dialysate flow.

The Molecular Adsorbents Recirculation System (MARS; Baxter, Unterschleißheim, Germany) is an extracorporeal liver dialysis system. It is a further development of albumin dialysis. The first step consists of albumin dialysis of the patient’s blood, whereby toxins are bound to the albumin in the dialysate circuit. In a second step, the albumin-containing dialysate is regenerated for recirculation. For this purpose, albumin-bound toxins are eliminated using an adsorber and by a second dialysis circuit.

In three of eight patients receiving liver replacement therapy, standard CVVHD with RCA was discontinued for the duration of MARS therapy. Furthermore, during MARS therapy, a different dialysis machine was used for liver dialysis (PRISMAFLEX system; Baxter). In addition, heparin was used for anticoagulation of the extracorporeal circuit according to the operating manual of the PRISMAFLEX system used.

### Metabolic complications of anticoagulation with citrate

For anticoagulation with citrate we used trisodium citrate (sodium citrate 4%; Fresenius Kabi, Bad Homburg, Germany). Because this contains an increased supply of sodium, we used multiBic (Fresenius Medical Care), a dialysate solution with a reduced degree of sodium, to avoid hypernatremia.

Accumulation of citrate and metabolic alkalosis are the main metabolic disorders described in the context of anticoagulation with citrate. Increased citrate plasma levels were detected by calculating the total tCa to iCa ratio (tCa/iCa) as described by Hetzel and colleagues [14]. A tCa/iCa ratio > 2.4 was considered a sign of accumulation of citrate.

The metabolism of 1 mol of citrate results in 3 mol of bicarbonate (HCO_3_^−^). Therefore, we used dialysate solutions with reduced HCO_3_^−^ content of 20 mmol/L (multiBic) to avoid development of metabolic alkalosis. Serum level of HCO_3_^−^, base excess, respiratory status (blood gas analysis) and pH value were determined at the same time points as iCa, at least four times per day. A blood pH value > 7.5 was considered as alkalosis.

### Statistical analysis

Continuous variables are expressed as the mean ± SD. Categorical variables are given as relative and absolute frequencies unless otherwise stated. The association between continuous variables was assessed by Spearman’s rank correlation testing.

To assess the influence of the laboratory variables on the development of accumulation of citrate or alkalosis, logistic and linear regression models were used. None of these complications was used as the reference category in the logistic regression. The predictive value of laboratory markers of liver function on the development of accumulation of citrate or alkalosis was assessed using ROC analysis. Statistical analysis was carried out using IBM SPSS version 20 software (IBM, Armonk, NY, USA).

## Results

**Fig. 1:**
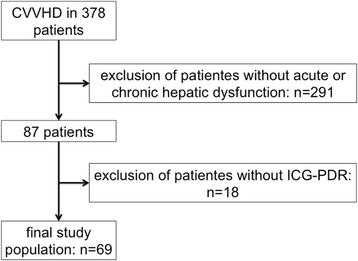
Flowchart displaying the study population

Between January 2009 and November 2012, a total of 378 patients underwent CRRT during the study period. Of these, 87 patients had acute or chronic liver dysfunction. Because ICG-PDR was not determined systematically, 69 patients were eligible for inclusion in this analysis (as shown in Fig. 1), resulting in a total of 1339 dialysis days of CRRT. The mean duration of CRRT was 19.4 days (SD, ±22.9; range, 1–105) (Table 1).

**Table 1:** Basic characteristics and outcomes illustrating liver dysfunction, severity of illness and outcome

Parameters	Results
Age, years	59.1 ± 12.4
Male sex, *n* (%)	47 (68.1)
BMI, kg/m^2^	26.5 ± 4.6
Pre-existing liver disease (double appointment is possible)	
Cirrhosis, *n* (%)	42 (60.9)
Hepatitis B or C, *n* (%)	10 (14.5)
Alcoholic cirrhosis, *n* (%)	20 (29)
Hepatocellular carcinoma, *n* (%)	9 (13)
Others, *n* (%)	22 (31.9)
MELD score at admission	19.7 ± 9.6
Severity of illness	
Maximum SAPS II score during stay at hospital	56.2 ± 13.6
Maximum TISS score during stay at hospital	24.2 ± 8.9
Sepsis during dialysis period, *n* (%)	29 (42)
Mechanical ventilation during stay in ICU, *n* (%)	57 (82.6)
Outcome	
Duration of ventilation, h	425.5 ± 507.8
Length of stay in hospital, days	62.9 ± 64.9
Length of stay in ICU, days	30.4 ± 31.8
Death, *n* (%)	40 (58.0)

*Abbreviations: BMI* Body mass index, *ICU* Intensive care unit, *MELD* Model for End-stage Liver Disease, *SAPS II* Simplified Acute Physiology Score II, *TISS* Therapeutic Intervention Scoring System

Results are shown as mean ± SD or as the number of patients and corresponding percentage with respect to all 69 patients

Most patients had pre-existing liver disease with a mean bilirubin at admission of 7.1 (SD, ±10.4; range, 0.2–41.6), mean ICG-PDR < 10% and mean Model for End-stage Liver Disease (MELD) score of 19.7. During the dialysis period, the average maximum serum level of bilirubin was 15.6 ± 11.4 mg/dl. Liver replacement therapy was performed in eight patients. Thirty patients received liver transplants. Because the aim of this study was to investigate the influence of liver function on the metabolism of citrate during long-term CRRT, the main focus was liver function at the beginning and during the course of dialysis, as shown in Table 2.

**Table 2:** Parameters of renal and liver function at start of CRRT and mean liver function during dialysis period

Parameters	At start of CRRT	During dialysis period
Creatinine, mg/dl	2.7 ± 1.3	
eGFR, CKD-EPI	24.7 ml/minute	
Cystatin C, mg/dl	3.2 ± 1.1	
GFR, cystatin C	17.7 ml/minute	
Bilirubin, mg/dl	9.5 ± 10.6	7.9 ± 6.8
Albumin, g/L	24.7 ± 7.0	25.0 ± 4.1
Cholinesterase, ×1000 IE/L	2.8 ± 1.7	2.6 ± 1.2
ICG-PDR	7.6 ± 5.8	9.9 ± 6.3
MELD score	29.8 ± 5.1	

*Abbreviations: CKD-EPI* Chronic Kidney Disease Epidemiology Collaboration equation, *CRRT* Continuous renal replacement therapy, *eGFR* Estimated glomerular filtration rate, *GFR* Glomerular filtration rate, *ICG-PDR* Indocyanine green plasma disappearance rate, *MELD* Model for End-stage Liver Disease, *IE/L* International Units per L

Results are shown as mean ± SD

At admission, most patients showed impaired renal function, and three patients were on chronic dialysis before admission (Table 2). The indications for dialysis were mainly hypervolaemia (46.4%), uraemia (43.5%) or hyperkalaemia (5.8%). The mean dialysis period was 19.4 days (SD, ±22.9).

### Anticoagulation with citrate

Regional anticoagulation with citrate was used in 99.1% of all 1339 CRRTs during the study period. All patients started as described in the “Methods” section with standard flow rates. In 40.6% of all patients and 48.5% of all dialysis performed, exclusively standard flow rates of blood and dialysate were applied during the whole duration of CRRT without a need for change owing to metabolic disorders. In three of eight patients receiving MARS for liver replacement therapy, anticoagulation was performed with heparin and not with citrate during this treatment period.

The effective mean blood flow was 96 ml/minute (SD, ±9; range, 80–138), and the mean dialysate flow was 2188 ml/h (SD, ±452; range, 1200–3600), resulting in an effective dialysis dose of 28.9 ml/h/kg. Sufficient anticoagulation was achieved with a mean dose of citrate of 4.0 mmol/L blood (SD, ±0.3; range, 3.4–4.7), resulting in a mean iCa of 0.30 mmol/L (SD, ±0.03; range, 0.12–0.38) in the extracorporeal circuit. The effectiveness of anticoagulation yielded a mean longevity of circuit filters of 62.2 h (SD, ±11.2; range, 24–72). To restore physiological serum levels of calcium, mean calcium substitution of 1.6 mmol/L dialysate (SD, ±0.3; range, 0.6–2.2) was given.

### Metabolic disorder due to anticoagulation with citrate

**Fig. 2:**
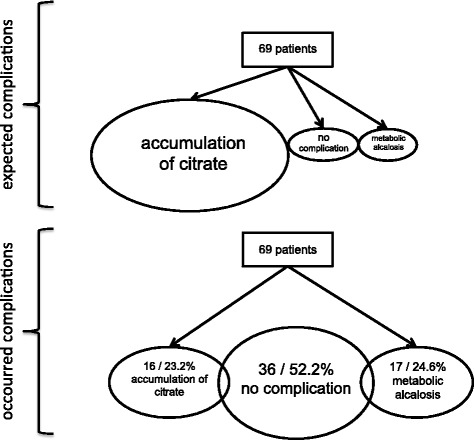
The occurrence of metabolic complications in this study compared with what would have been expected in patients with impaired liver function undergoing continuous renal replacement therapy with regional citrate anticoagulation. *CVVHD* Continuous venovenous haemodialysis; *ICG-PDR* Indocyanine green plasma disappearance rate

The occurrence of metabolic complications in this study compared with what would have been expected in patients with impaired liver function undergoing CRRT with RCA is shown in Fig. 2. In 16 patients (23.2%), a ratio of tCa/iCa > 2.4 occurred during CRRT, indicating accumulation of citrate. Mean tCa during the dialysis period was 2.54 ± 0.17 mmol/L. In nine patients, tCa was > 2.7 mmol/L. The highest serum calcium level of tCa measured was 3.7 mmol/L in one patient at 1 day. At any time clinical complications occurred due to hyper- or hypocalciemia. Hypercalcaemia or accumulation of citrate did not require that RCA be stopped in any patient.

Mean HCO_3_^−^ was 25.8 ± 4.4 mmol/L during the dialysis period. Metabolic alkalosis occurred in 17 patients (24.6%). Maximal HCO_3_^−^ ranged from 19 to 46 mmol/L, accompanied by a mean base excess of 7.7. Adequate modification of CRRT resolved hypercalcaemia or alkalosis.

### Factors influencing accumulation of citrate

Comparing the 16 patients showing signs of citrate accumulation with the remaining 53 patients, we found that MELD score at the start of dialysis was higher (33.1 ± 4.2 versus 28.8 ± 5.0; *p* = 0.001), Quick value was lower (41.2 ± 23.6 versus 57.1 ± 19.3; *p* = 0.004), duration of dialysis was shorter (11.6 ± 8.6 versus 22.1 ± 26.3; *p* = 0.014) and dose of citrate per litre of blood was higher (4.2 ± 0.6 versus 4.0 ± 0.6; *p* = 0.025). MARS for liver replacement therapy had no impact on accumulation of citrate, as shown in Table 3.

**Table 3:** Patients with and without citrate accumulation, hepatic function at start of continuous renal replacement therapy

Parameter	Accumulation of citrate; tCa/iCa ratio > 2.4	No accumulation of citrate; tCa/iCa ratio ≤ 2.4	*p* Value
No. of patients	16	53	
Age, years	57.4 ± 14.0	59.6 ± 11.9	n.s.
MELD score	33.1 ± 4.2	28.8 ± 5.0	0.001
Prothrombin time, Quick value (%)	41.2 ± 23.6	57.1 ± 19.3	0.004
Bilirubin, mg/dl	8.3 ± 11.1	8.7 ± 11.9	n.s.
ICG-PDR	7.4 ± 5.5	10.7 ± 6.4	n.s.
Duration of CRRT, days	11.6 ± 8.6	22.1 ± 26.3	0.014
Mean flow of dialysate, ml/h	2234.1 ± 501.9	2173.7 ± 437.8	n.s.
Effective dialysis dose, ml/kg/h	28.8 ± 8.5	28.9 ± 8.6	n.s.
Mean dose of citrate, mmol/L blood	4.2 ± 2.6	4.0 ± 2.6	0.025
Mean Ca^2+^ substitution, mmol/L dialysate	1.66 ± 0,15	1.55 ± 0,30	0.038
Total Ca^2+^, mmol/L	2.67 ± 0.17	2.47 ± 0.15	0.000
iCa post–filter, mmol/L	0.32 ± 0.02	0.29 ± 0.05	0.001
iCa in patient, mmol/L	1.07 ± 0.06	1.14 ± 0.09	0.000
Death, *n* (%)	11 (68.8%	29 (54.7%	n.s.
Vasopressor support, % of CRRT duration	65.8 ± 39.7	55.2 ± 32.8	n.s.
Maximal SAPS II score	56.2 ± 14.4	56.2 ± 13.5	n.s.
Stay in ICU, days	14.9 ± 11.6	35.1 ± 34.1	0.001

*Abbreviations: CRRT* Continuous renal replacement therapy, *iCa* Ionised serum calcium, *ICG-PDR* Indocyanine green plasma disappearance rate, *ICU* Intensive care unit, *MELD* Model for End-stage Liver Disease, *n.s.* Not significant, *SAPS II* Simplified Acute Physiology Score II, *tCa/iCa* Total serum calcium/ionised serum calcium

Results are shown as mean ± SD

In correlation analysis only MELD score and prothrombin time (Quick value) at the start of dialysis showed a weak correlation (0.303 and −0.387, respectively; *p* = 0.001). In a binary logistic regression model only prothrombin time showed a small impact on the accumulation of citrate. A reduction of 1% in Quick value resulted in an elevation of 0.004 of the tCa/iCa ratio. In ROC analysis the predictive capacity of hepatic parameters (bilirubin, CHE, prothrombin time [Quick value] and ICG-PDR), the dose of citrate and the ICU length of stay for the accumulation of citrate were tested. The resulting AUCs were 0.253 to 0.662, respectively.

### Factors influencing development of alkalosis

Comparing the 17 patients showing signs of alkalosis with the remaining 52 patients, the Quick value was higher (74.0 ± 11.7 versus 56.4 ± 18.8; *p* = 0.000), the ICG-PDR was higher (13.4 ± 5.6 versus 9.0 ± 6.2; *p* = 0.029), the dose of citrate per litre of blood was lower (3.8 ± 0.2 versus 4.1 ± 0.3; *p* = 0.000), the flow of dialysate was lower (1935 ± 267 versus 2270 ± 470 ml/h; *p* = 0.001) and mortality was lower (35.3% versus 65.4%; *p* = 0.029).

In correlation analysis ICG-PDR and prothrombin time (Quick value) showed a weak correlation with the development of alkalosis (0.348 and 0.427; *p* = 0.029 and 0.000, respectively). In a binary logistic regression model only prothrombin time (Quick value) and ICG-PDR showed a weak impact on the development of alkalosis (0.072 and −0.003, respectively). In ROC analysis the predictive capacity of hepatic parameters (bilirubin, CHE, prothrombin time [Quick value] and ICG-PDR) for the development of alkalosis was tested. Only prothrombin time and ICG-PCR showed AUCs above 0.70 (0.785 and 0.736, respectively).

## Discussion

In this retrospective evaluation, we demonstrate that in critically ill patients with severe hepatic dysfunction, regional anticoagulation with citrate for CRRT is possible, even for long-term dialysis. Although patients showed pronounced impaired liver function, citrate accumulation occurred in only one of four patients and was far less dramatic than expected.

Under physiological conditions, citrate is metabolised by the liver and to a lesser extent by the skeletal muscle [5]. Therefore, RCA is considered as contraindicated in cases of impaired liver function resulting in accumulation of citrate [6, 15–18]. Increased citrate plasma levels are indirectly detected, as described by Hetzel and colleagues [14], through an increasing ratio of total serum calcium (tCa/iCa). The limit of this ratio varies in the literature from 2.1 to ≥ 2.5 [19–21]. Under physiological conditions, tCa ranges from 2.2 to 2.6 mmol/L and iCa ranges from 0.90 to 1.20 mmol/L. The resulting ratio (tCa/iCa) ranges from 1.8 to 2.4. Thus, a tCa/iCa ratio > 2.4 was considered a sign of accumulation of citrate. This threshold was also previously chosen by Link and colleagues [21]. Although all patients included showed pronounced impaired liver function, in almost half of these patients any aspect of metabolic or electrolyte disturbance occurred, and only one of four had signs of citrate accumulation. Furthermore, slight changes in the dialysate and blood flows mostly allowed correction of looming signs of metabolic disorders. These results are in contrast to the conventional wisdom in the literature. However, there is anecdotal evidence that the use of citrate in patients with impaired liver function would be possible [3, 22, 23]. Of note, some studies have reported RCA after liver transplant [8, 24]. However, liver function normally improves after transplant. Furthermore, the described mean dialysis periods were quite short at 5–8 days [8, 24].

In assessing metabolic complications of citrate anticoagulation, in particular the duration of dialysis is of central importance because in prolonged CRRT high cumulative doses of citrate are administered. However, in the literature, only short dialysis periods are described [3, 8, 23–26]. The recently published liver citrate anticoagulation threshold (L-CAT) trial convincingly showed the safety of CRRT-RCA in patients with severely impaired liver function [9]. Of note, the reported observation period included the first 72 h of CRRT-RCA treatment. In contrast, we report the whole duration of CRRT-RCA; the mean duration of the dialysis period was 19 days, resulting in high cumulative doses of citrate.

Taken together, hepatic function seems not to be exclusively or predominantly responsible for citrate metabolism. We therefore hypothesise that in critically ill patients with impaired liver function a part of citrate metabolism is shifted into muscle cells or any other cells. This also would explain why the duration of dialysis with consecutive high cumulative doses of citrate does not result in accumulation and metabolic disorder.

Theoretically, smaller doses of citrate would also reduce the cumulative dose during long-term dialysis and RCA with citrate. However, adequate anticoagulation is an essential requirement to ensure the patency of the dialysis circuit. Therefore, mean circuit lifespan is an indirect indicator of the effectiveness of anticoagulation. RCA circuit lifespans up to 48 h and more have been reported [14, 25, 27, 28]. In our study mean circuit patency was 62.2 h, confirming adequate anticoagulation over time. Thus, despite high cumulative doses of citrate during a mean dialysis period of 19 days, citrate accumulation occurred in only 23% of patients.

Metabolism of administrated citrate can induce metabolic alkalosis. Under physiological conditions citrate is cleared by the citric acid cycle (tricarboxylic acid cycle), resulting in 3 mmol of NaHCO_3_^−^ per 1 mmol of trisodium citrate [29]. In the literature the incidence of alkalosis during RCA with citrate is reported in 23–55% of patients [30, 31]. Different citrate formulations and HCO_3_^−^ concentrations of the dialysate or replacement solutions may partially explain these different incidences of alkalosis. However, the occurrence of alkalosis also depends on rapid metabolism of citrate. This is thought to be dependent mainly on liver function [8]. In one-fourth of patients in our study, metabolic alkalosis occurred, in line with other reported incidences. Of note, in contrast to studies reporting on alkalosis, all of our included patients had severely impaired liver function. We interpret this fact as a further indication of our former described hypothesis that an adequate citrate metabolism must be possible also outside the liver.

In the literature, citrate metabolism is primarily associated with hepatic function. Therefore, baseline liver function parameters should be predictive regarding citrate accumulation during CRRT with RCA. However, most standard laboratory liver function parameters showed poor predictive capabilities [3, 6, 26]. This raises the question whether impaired hepatic function is really the main reason for accumulation of citrate. This poor prediction of citrate accumulation by parameters of liver function appears to be a further indication that an effective extrahepatic metabolism of citrate seems to exist. Our results are consistent with the recently published study by Slowinski and colleagues [9]. However, the reported observation period of CRRT was 3 days (first 72 h of CRRT). Thus, comparability with our study is limited.

Because in all cells citrate can be metabolised within the Cori cycle (tricarboxylic acid cycle), we hypothesise that an impaired perfusion on the microvascular and cellular levels is critical to the metabolism of citrate. This hypothesis is supported by the observations of Schultheiss and colleagues, who described a baseline serum lactate level ≥ 3.4 mmol/L as a predictor of citrate accumulation [3], and of Link and colleagues, who showed that in patients with signs of citrate accumulation, mean arterial blood pressure was lower and dose of norepinephrine was higher than in those without citrate accumulation [21]. This hypothesis has also been suggested by recent studies, some of them pointing at hyperlactataemia [32] or, more specifically, at lactate kinetics during treatment [33] as a useful predictor for citrate accumulation. Disturbed microvascular circulation could also cause altered hepatic function, resulting in an association of impaired liver function and citrate accumulation. This hypothesis would also explain why in the literature inconsistent results exist concerning liver function and accumulation of citrate. Moreover, a correlation was described between the tCa/iCa ratio and hepatic clearance measured by the ICG-PDR and multi-organ dysfunction measured by SAPS II score during CRRT-RCA [21]. On the basis of our data, we cannot prove the hypothesis that shock and disturbed microcirculation are mainly responsible for disturbed citrate metabolism. However, patients developing citrate accumulation had a higher need for vasopressor therapy and showed lower ICG-PDR. In addition, reduced ICG-PDR is not only a sign of impaired liver function but also was recently shown to be correlated with impaired hepatic perfusion [34].

### Limitations

The retrospective design of this study limits the meaningfulness of our hypothesis. The number of included patients is quite small. This could result in bias despite the long dialysis period and the high number of dialysis. Furthermore, ICG-PDR is not available in all centres and was not performed in all patients in our centre. Because the duration of MARS results in discontinuity of the load with citrate, this may have an impact on metabolic complications. Not all laboratory data are available in all patients at all time points desired for this evaluation.

## Conclusions

Our findings may serve to somewhat dispel the notions that RCA is contraindicated in critically ill patients with impaired liver function. Citrate metabolism seems not to be restricted to the liver. Therefore, liver failure in patients treated by CRRT with RCA does not automatically result in accumulation of citrate. However, caution and close monitoring of metabolic disorders are needed because wrong management of RCA can result in serious adverse effects in critically ill patients with impaired liver function. Further studies are warranted to confirm our findings.

## Abbreviations

BMI: Body mass index
CHE: Cholinesterase
CKD-EPI: Chronic Kidney Disease Epidemiology Collaboration equation
CRRT: Continuous renal replacement therapy
CRRT-RCA: Continuous renal replacement therapy with regional citrate anticoagulation
CVVHD: Continuous venovenous haemodialysis
eGFR: Estimated glomerular filtration rate
HCO_3_^−^: Bicarbonate
ICG-PDR: Indocyanine green plasma disappearance rate
ICU: Intensive care unit
L-CAT: Liver citrate anticoagulation threshold
MARS: Molecular Adsorbents Recirculation System
MELD: Model for End-stage Liver Disease
n.s.: Not significant
RCA: Regional citrate anticoagulation
RRT: Renal replacement therapy
SAPS II: Simplified Acute Physiology Score II
tCa/iCa: Total serum calcium/ionised serum calcium
TISS: Therapeutic Intervention Scoring System
